# Typho-Diplococcaemia

**Published:** 1916-10

**Authors:** 


**Typho-Diplococcaemia.** A. Sartory, L. Spillman and Ph. Lasseur, Bull. de la Soc. de Med. de Nancy, 1916, page 611, have described a diplococcus frequently found in large numbers in the blood of patients suspected of typhoid or paratyphoid. Some others have verified their conclusion, Tremolieres has observed 16-17% of this infection (among cases of clinical typhoid). The organism is definite and acts alone to imitate typhoid or paratyphoid.

